# Decoupled Online-Inertia-Tunable Robotic Fish: Design, Modeling and Performance Analysis

**DOI:** 10.3390/biomimetics11100695

**Published:** 2026-10-01

**Authors:** Jinan Yu, Jian Wang, Xinyu Cui, Peng Li, Sai Deng, Xiangyu Sun, Zhengxing Wu

**Affiliations:** 1 The Key Laboratory of Cognition and Decision Intelligence for Complex Systems, Institute of Automation, Chinese Academy of Sciences, Beijing 100190, China; jinan.yu@ia.ac.cn (J.Y.); jianwang@ia.ac.cn (J.W.); lipeng2015@ia.ac.cn (P.L.); sai.deng@ia.ac.cn (S.D.); sunxiangyu2023@ia.ac.cn (X.S.); 2 School of Artificial Intelligence, University of Chinese Academy of Sciences, Beijing 100049, China; cuixinyu2021@ia.ac.cn; 3 State Key Laboratory of Robotics and Intelligent Systems, Shenyang Institute of Automation, Chinese Academy of Sciences, Shenyang 110169, China; 4 State Key Laboratory of Autonomous Intelligent Unmanned Systems, Beijing Institute of Technology, Beijing 100081, China

**Keywords:** bionic robots, impedance adjustment, robotic fish, online-tunable inertia

## Abstract

Fish can actively modulate the mechanical properties of their muscles to adapt to complex hydrodynamic environments, and extensive research has been dedicated to replicating such adaptive locomotion capabilities in bionic robotic fish. Unlike the widely investigated variable-stiffness tail designs in existing studies, this paper proposes a decoupled tunable rotational inertia mechanism for the robotic fish caudal peduncle, which enables independent regulation of tail rotational inertia without altering the global center of mass of the robot. A symmetric cable-driven transmission system equipped with a pair of oppositely moving mass spheres is adopted to realize real-time online inertia tuning while sustaining the robot’s dynamic balance. A high-fidelity fluid-structure interaction (FSI) dynamic model is established by integrating the pseudo rigid body method (PRBM) and the boundary data immersion method (BDIM). Simulation and experimental results demonstrate that rotational inertia modulation exerts a significant regulatory effect on the swimming performance of bionic robotic fish. Specifically, increased rotational inertia enhances thrust generation and forward swimming velocity within a specific tail-beat frequency range, whereas it degrades locomotion performance when the tail-beat frequency exceeds a critical threshold. Remarkably, the performance improvement enabled by enhanced rotational inertia can only be achieved via internal mass redistribution. In contrast, directly attaching additional mass to increase rotational inertia will almost invariably impair the swimming capability of the robot. This study reveals the unique regulatory effect of rotational inertia as an independent control input for bio-inspired underwater robots, providing a unique regulatory effect for the development of unified impedance-inertia adaptive control frameworks for future bionic underwater platforms.

## 1. Introduction

Fish-inspired robotic systems realize high-efficiency and high-maneuverability underwater locomotion via dynamic body-fluid interactions [1,2,3,4]. Among the diverse determinants of propulsion performance, the mechanical characteristics of the caudal peduncle serve as a critical parameter that governs thrust generation, energy efficiency, and wake evolution for fish adopting the body and/or caudal fin (BCF) swimming mode [5,6,7,8]. In nature, real fish can actively modulate the mechanical responses of their musculoskeletal systems, thereby adaptively adjusting to variable swimming speeds and complex aquatic environments [9,10,11]. Such excellent adaptive regulation characteristics offer profound inspiration for the structural design and motion control of the bionic robotic fish.

To replicate these sophisticated adaptive locomotion capabilities, many bionic robotic fish are commonly equipped with compliant tails featuring tunable stiffness, which has driven extensive investigations into their swimming efficiency, maneuverability and overall hydrodynamic performance [12,13,14,15]. Micklem et al. [16] adjusted the longitudinal profile and cross-sectional area of a robotic fish tail to modulate its moment of inertia. Hu et al. [17] developed a programmable real-time online stiffness modulation scheme that synchronously regulates body stiffness with tail-beat cycles and swimming frequencies to comprehensively optimize thrust, velocity, and propulsive efficiency. Wang et al. [18] adopted segmented variable-stiffness artificial muscles imitating fish muscles to reconstruct anguilliform, carangiform, and thunniform swimming gaits, revealing the evolutionary principle of adaptive swimming relying on real fish muscle stiffness adjustment. Meanwhile, Wolf et al. [19] regulated tail stiffness via the antagonistic action of hydraulic or pneumatic pressure, where cyclic pressure changes simultaneously generated the propulsive force for tail flapping.

Nevertheless, nearly all existing solutions are confined to the regulation of structural stiffness or damping. As one of the three core mechanical parameters determining system dynamic characteristics, inertia modulation has been drastically understudied in prior research, which creates a prominent research gap and great potential for performance enhancement of fish-inspired underwater robots.

Variable inertia regulation has been widely adopted to optimize gait patterns and stabilize attitude across multiple robotic platforms, including quadruped robots, industrial manipulators and intelligent ground mobile robots [20,21]. Hong et al. [22] introduced an internal eccentric rolling mass module to actively redistribute the longitudinal moment of inertia of autonomous underwater vehicles (AUVs). Benefiting from this mechanism, roll control torque can be produced independent of forward cruising velocity, which effectively suppresses low-speed rolling oscillation and stabilizes vehicle posture to satisfy the operational demands of sonar detection missions. Wang et al. [23] designed a retractable variable-inertia tail structure that adjusts the overall rotational inertia via rearrangement of tail-mounted masses. The generated inertial torque further optimizes the dynamic behaviours during jumping and landing phases of quadruped robots and avoids unexpected pitching tumbling. Zhou et al. [24] rearranged the principal inertia axes of floating dual-arm systems via joint coordination, achieving propellant-free target detumbling through inertia redistribution and ameliorating post-capture multi-body coupling dynamics. Xu et al. [25] optimized arm mass layout using lattice topology to fully tune coupled joint inertia, weakening manipulator-induced inertial perturbations to the drone body and facilitating high-precision aerial grasping. Considering that the hydrodynamic performance of bionic robotic fish is strongly coupled with its undulatory kinematics, and the oscillatory deformation of flexible tails under fluid–structure interaction is highly sensitive to system dynamic parameters, inertial tuning is reasonably inferred to exert a substantial effect on the swimming performance of fish-inspired underwater robots.

Inspired by these observations, this paper investigates the effects of inertia as an independent variable on the swimming performance of bionic robotic fish. Compared with existing studies, the main contributions of this paper are summarized as follows:

(1) A decoupled bionic robotic fish capable of real-time online inertia regulation is designed and prototyped. Benefiting from a symmetric cable-driven actuation system, the proposed platform realizes independent inertial tuning while keeping the overall center of mass fixed throughout adjustment.
(2) A high-precision fluid–structure interaction (FSI) dynamic model for the robotic fish is established by combining the pseudo-rigid body method (PRBM) and boundary data immersion method (BDIM).
(3) Systematic experiments and analyses are carried out to characterize the frequency-dependent influences of rotational inertia on locomotion performance. Quantitative results demonstrate that elevated inertia does not universally degrade swimming capability; under designated working conditions, appropriate inertia augmentation can effectively improve thrust output and cruising velocity.

The remainder of this paper is organized as follows. Section 2 elaborates on the mechanical design of the decoupled variable-inertia mechanism. Section 3 establishes the dynamic model of the robotic fish system in detail. Section 4 presents the numerical simulation analyses and physical experimental validations. Section 5 concludes this work and outlines potential directions for future investigations.

## 2. Development of the Decoupled Online-Inertia-Tunable Robotic Fish

**Figure 1:**
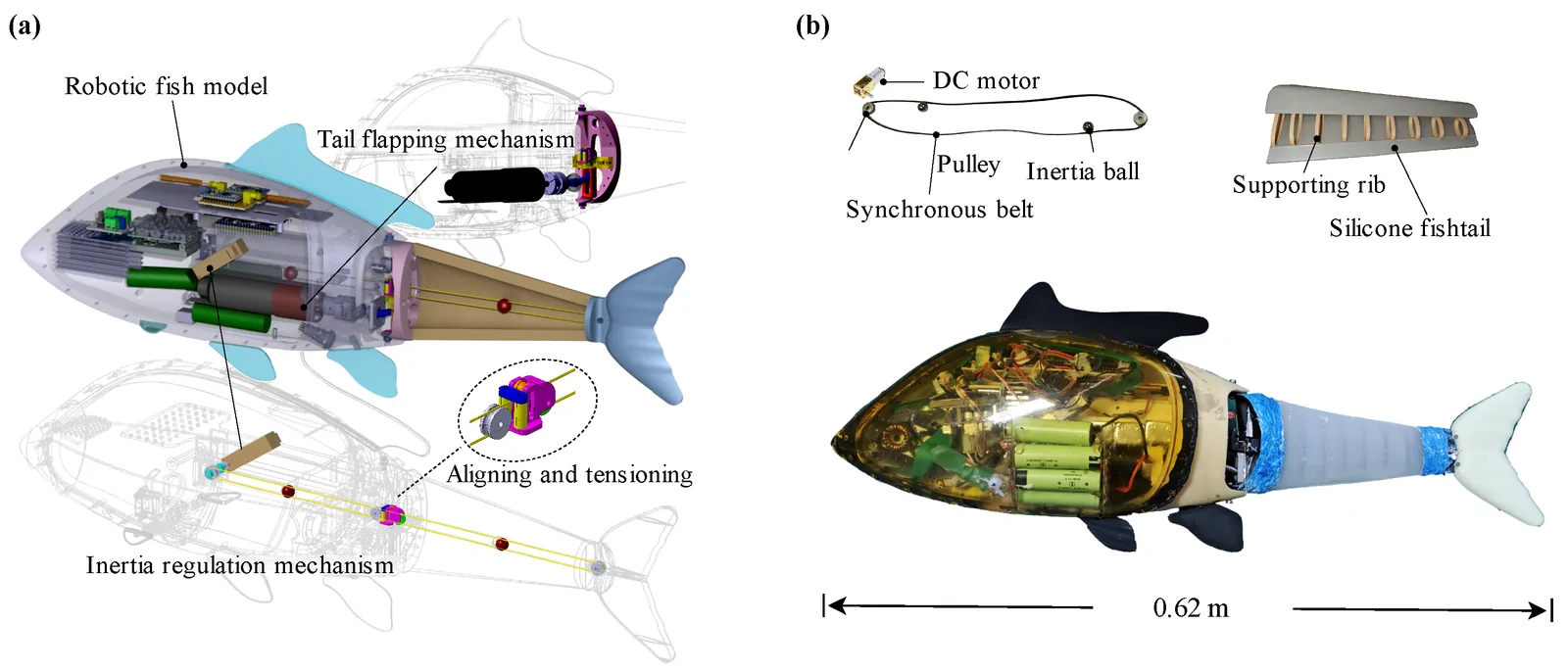
Decoupled online-inertia-tunable robotic fish demonstration. (**a**) Design of the robotic fish. (**b**) Robotic fish prototype.

Taking the morphological characteristics of common carp as the bionic prototype, we develop a bionic robotic fish equipped with one active driving joint matched with a passively flexible caudal peduncle, as illustrated in Figure 1. Apart from conventional power supply, control system and wireless communication units, the robotic fish is embedded with a specially designed decoupled real-time inertia modulation system. Specifically, the inertia tuning system mainly consists of a DC servo motor, a timing belt, matched driving and driven pulleys, and two movable mass spheres. The entire belt–pulley transmission system is laid out in a mirror-symmetric layout relative to the swing axis of the caudal fin. Two identical mass spheres are mounted symmetrically on the upper and lower segments of the timing belt and initially positioned equidistantly from the flapping axis. Actuated by the DC motor, the two spheres move synchronously towards or away from each other with identical displacement magnitudes. Such symmetrical translational motion thoroughly eliminates overall center-of-mass (COM) drift, enabling decoupled, real-time adjustment of the system’s rotational inertia. Moreover, an integrated positioning and tensioning assembly is mounted at the midpoint of the timing belt to guarantee steady and durable operation under high-frequency reciprocating flapping. In order to actuate periodic tail flapping, a compact spatial crank–rocker linkage is deployed. To fit the confined internal installation space inside the robotic fish hull, the tail actuation mechanism is arranged beneath the body’s longitudinal central axis. This linkage transforms the unidirectional continuous rotation of the driving motor into reciprocating swing motion of the bionic tail. The flexible caudal peduncle adopts a hollow composite structure assembled from elastic silicone matrix and a rigid load-bearing support skeleton. Key electromechanical specifications of major components of the robotic fish are tabulated in Table 1.

## 3. Modeling of the Robotic Fish

### 3.1. Kinematics

**Figure 2:**
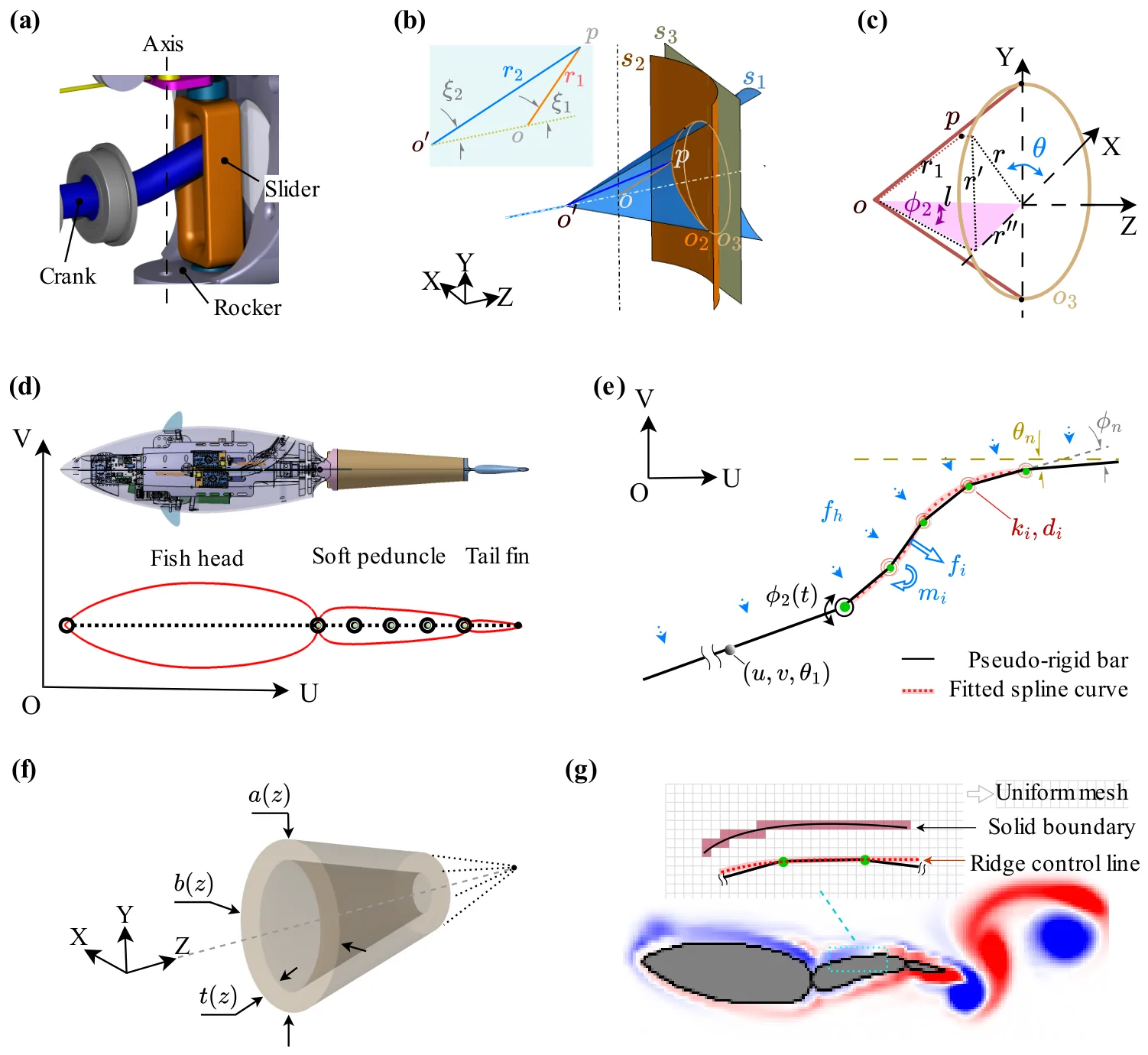
Schematic diagrams of robotic fish modeling. (**a**) Drive mechanism diagram. (**b**) Spatial constraint representation of the space crank-slider contact point. (**c**) Geometric analysis of the driving kinematics. (**d**) PRBM description. (**e**) Parameter schematic of the PRBM. (**f**) Structure diagram of the flexible caudal peduncle. (**g**) Schematic of the FSI dynamics.

Figure 2a presents the detail of the spatial crank mechanism responsible for the reciprocating tail flapping. The corresponding geometric abstraction of this mechanism is illustrated in Figure 2b, where $o^{\prime }$ denotes the pivot center of the crank, *o* the swing center of the rocker, and *p* the point of contact between the crank and the slider. Under the geometric constraints $s_{1}$ and $s_{2}$, the trajectory of point *p* traces a spatial curve $o_{2}$, whose orthogonal projection onto plane $s_{3}$ yields a planar curve $o_{3}$. Denote $r_{1}$ and $r_{2}$ as the lengths of segments op and $o^{\prime }p$, respectively. It is noteworthy that $r_{1}$ and $r_{2}$ are not constant, owing to the complex three-dimensional trajectory (x,y,z) of point *p*. Based on basic trigonometric geometry, the relationship between $\xi_{1}$ and $\xi_{2}$ is as follows:

(1) $$r_{2}\cdot \sin\xi_{2}=r_{1}\cdot \sin\xi_{1}$$

In Figure 2c, *l* denotes the vertical distance from point *o* to the projection plane $s_{3}$, and *r* is the projected length of $r_{1}$ onto $s_{3}$, with its *X*- and *Y*-axis components represented by $r^{\prime }$ and $r^{\prime \prime }$, respectively. Here, $\phi_{2}$ denotes the swing angle of the fishtail, and θ corresponds to the rotational angle of the driving motor. From the geometric constraints, the following expressions are obtained:

(2) $$\tan\phi_{2}=\frac{r^{\prime \prime }}{z}=\cos\theta \cdot \tan\xi_{1}$$

By combining Equations (1) and (2), the functional mapping between the motor rotation angle θ and the fishtail swing angle $\phi_{2}$ can be expressed as:

(3) $$\phi_{2}=\arctan\left(\cos\theta \cdot \tan\left(\arcsin\left(r_{2}\cdot \sin\xi_{2}/r_{1}\right)\right)\right)$$

In this study, the pseudo-rigid-body model (PRBM) is adopted to establish the dynamic model of the bionic robotic fish [5], wherein the whole robot is discretized into six interconnected rigid segments. The foremost segment corresponds to the fish head, while the rearmost segment acts as the caudal fin; the remaining four intermediate segments collectively approximate the deformation characteristics of the flexible caudal peduncle, as illustrated in Figure 2d. The position vectors of each segment’s centroid in the global coordinate frame and the deflection angle of each segment are defined as $r_{i}$ and ${\dot{\theta }}_{i}$, respectively.

(4) $$r_{i}=\left\{\begin{array}{cc} r_{l,i}, & i=1\\ r_{t,1}+\sum_{k=2}^{i-1}r_{k}+r_{l,i}, & i>1 \end{array}\right.$$

(5) $$\theta_{i}=\sum_{k=1}^{i}\phi_{k}$$

where $r_{l,i}$ represents the position vector from the proximal joint to the centroid of the *i*-th link. $r_{t,i}$ denotes the position vector from the centroid of the *i*-th link to its distal joint (e.g., $r_{t,1}$ denotes the vector from the centroid of the first link to the first joint). $\phi_{k}$ stands for the relative rotation angle between the *k*-th link and the (k−1)-th link, with $\phi_{1}=\theta_{1}$.

The generalized coordinates q are defined as the pose $\left(u,v,\theta_{1}\right)$ of the first link in the horizontal plane, along with the relative joint angular displacements: $q={\left[u,v,\theta_{1},\phi_{3},\phi_{4},\phi_{5},\phi_{6}\right]}^{⊤}$. The corresponding velocity mappings from the generalized coordinates to the operational space are given by

(6) $${\dot{r}}_{i}=J_{v,i}\left(q\right)\cdot \dot{q}\;,\;\;\;\;\;J_{v,i}\in R^{2\times n}$$

(7) $${\dot{\theta }}_{i}=J_{\omega ,i}\left(q\right)\cdot \dot{q}\;,\;\;\;\;\;J_{\omega ,i}\in R^{1\times n}$$

where $J_{v,i}\left(q\right)$ and $J_{\omega ,i}\left(q\right)$ correspond to the Jacobian matrices for linear velocity and angular velocity, respectively.

### 3.2. FSI Dynamics

The dynamic model of the proposed robotic fish is established using the Lagrangian mechanics formulation. Given that the robotic fish performs purely planar locomotion, the total kinetic energy of the system can be formulated as the superposition of translational and rotational kinetic energy components contributed by all segmented links:

(8) $$T=\frac{1}{2}\sum_{i=1}^{n}\left(m_{i}{\dot{r}}_{i}^{⊤}{\dot{r}}_{i}+I_{i}{\dot{\theta }}_{i}^{2}\right)$$

where $m_{i},I_{i}$ denote the mass and rotational inertia of each link, respectively. The total potential energy of the system consists of the elastic potential energy stored in the kinematic joints:

(9) $$U=\frac{1}{2}\sum_{i=1}^{n}\left(k_{i}\phi_{i}^{2}\right)$$

where $k_{i}$ represents the spring stiffness coefficient of each joint.

Thus, the Lagrange equation of the robotic fish system is formulated as:

(10) $$\frac{d}{dt}\left[\frac{\partial (T-U)}{\partial \dot{q}}\right]-\frac{\partial (T-U)}{\partial q}=Q_{f_{\mathrm{nc}}}$$

where $Q_{f_{\mathrm{nc}}}$ denotes the vector of non-conservative generalized forces. For the system under consideration, this non-conservative generalized force vector consists of three components: the motor actuation forces $J_{\omega }^{⊤}f_{\mathrm{drive}}$, the hydrodynamic forces $J_{v}^{⊤}f_{\mathrm{hydro}}$ exerted by the surrounding environment, and the damping forces $J_{\omega }^{⊤}f_{\mathrm{damp}}$ arising from the joints and the flexible caudal peduncle.

Combining the above expressions yields the final dynamic equation:

(11) $$M\left(q\right)\ddot{q}+C\left(q,\dot{q}\right)\dot{q}+G\left(q\right)=J_{v}^{⊤}f_{\mathrm{hydro}}+J_{\omega }^{⊤}f_{\mathrm{damp}}+J_{\omega }^{⊤}f_{\mathrm{drive}}$$

where $M\left(q\right),C\left(q,\dot{q}\right),G\left(q\right)$ represent the inertia matrix, the Coriolis-centrifugal terms and the conservative generalized forces, respectively.

Next, we derive the mapping between the flexibility of the caudal peduncle and the equivalent torsional stiffness of the joints in the PRBM framework. The proposed caudal peduncle is designed as a hollow structure with a variable elliptical cross-section (Figure 2f), and its bending stiffness can be expressed as:

(12) $$B=\frac{1}{4}E\pi \left[a\left(z\right)b{\left(z\right)}^{3}-\left(a\left(z\right)-t\left(z\right)\right){\left(b\left(z\right)-t\left(z\right)\right)}^{3}\right]$$

where a(z),b(z),t(z) represent the major-axis length, minor-axis length, and wall thickness of the elliptical cross-section at position *z*, respectively, and *B* denotes the bending stiffness. To approximate the morphology of a real fish while reducing system complexity, we assume the geometric parameters vary linearly along the caudal peduncle: $a\left(z\right)=k_{a}z$, $b\left(z\right)=k_{b}z$, and $t\left(z\right)=k_{c}z$. Accordingly, the bending stiffness at the *i*-th joint in the PRBM model is approximated by the following two-parameter form:

(13) $$k_{i}=K_{k}\cdot i^{4}+K_{b}$$

where $K_{k}$ and $K_{b}$ are the coefficients determining the torsional stiffness distribution along the caudal peduncle.

For damping forces, no explicit damping elements are included in the system. Instead, damping effects arising from joint friction and internal material dissipation are approximated using a linear damping model:

(14) $$f_{\mathrm{damp}}\left(i\right)=\left\{\begin{array}{cc} -D_{i}{\dot{q}}_{i}, & i\neq 1,2\\ 0, & i=1,2 \end{array}\right.$$

Regarding driving forces, position-based actuation is adopted in this work: the actuated joint (i.e., $\phi_{2}$) follows a prescribed trajectory. Consequently, the driving torque does not appear explicitly in the dynamic equations; rather, it induces equivalent inertial coupling forces on the other degrees of freedom. The generalized driving force can be expressed as:

(15) $$f_{\mathrm{drive}}\left(i\right)=M_{fd}{\ddot{\phi }}_{2}$$

where

$$M_{fd}=\sum_{i=1}^{6}\left[\left(m_{i}+\delta m_{i}\right)J_{v,i}^{⊤}\frac{\partial r_{i}}{\partial {\dot{\phi }}_{2}}+\left(I_{i}+\delta I_{i}\right)J_{\omega ,i}^{⊤}\frac{\partial \theta }{\partial {\dot{\phi }}_{2}}\right]$$

For hydrodynamic force computation, two approaches are adopted. The first employs empirical formulations based on the Morison equation:

(16) $$f_{\mathrm{hydro},i}=f_{\mathrm{hydro}⊥,i}\left(s\right)+f_{\mathrm{hydro}\|,i}\left(s\right)+f_{\mathrm{hydro}⊢,i}\left(s\right)$$

where

(17) $$f_{\mathrm{hydro}⊥,i}\left(s\right)=\int_{0}^{L_{i}}-\frac{1}{2}\rho C_{d}D_{i}v_{⊥,i}^{2}\left(s\right)\cdot {\hat{w}}_{i}\;ds$$

(18) $$f_{\mathrm{hydro}\|,i}\left(s\right)=\int_{0}^{L_{i}}-\frac{1}{2}\rho C_{f}D_{i}v_{\|,i}^{2}\left(s\right)\cdot {\hat{u}}_{i}\;ds$$

(19) $$f_{\mathrm{hydro}⊢,i}\left(s\right)=\int_{0}^{L_{i}}-\frac{1}{4}\pi \rho C_{a}D_{i}^{2}v_{⊥,i}\left(s\right)\cdot {\hat{w}}_{i}\;ds$$

where $s\in [0,L_{i}]$ is the coordinate along the *i*-th link; ${\hat{w}}_{i}$ and ${\hat{u}}_{i}$ are the unit normal and tangential vectors, $v_{⊥,i}$ and $v_{\|,i}$ are the corresponding scalar values; ρ is the fluid density; $C_{d},C_{f},C_{a}$ denote the pressure drag coefficient, the viscous resistance coefficient, and the added-mass force coefficient, respectively; $D_{i}$ represents the height of the vertical projection of the wetted surface.

The second approach employs the BDIM for numerical computation in the flow field [26,27]. As illustrated in Figure 2g, the 2D profile of the robotic fish is discretized on Cartesian grids. This approach combines the solid domain, fluid domain, and interface smoothing layer into a single global computational field. The outer contour and velocity field of the caudal peduncle are updated via normal offsetting, generating a smooth, flexible tail profile that dynamically adapts to the robotic fish’s real-time motion. After the caudal peduncle deforms, the fluid-solid coupling interface of BDIM receives the updated velocity field for the subsequent fluid computation. The hydrodynamic forces computed from the interface smoothing layer adjacent to the fish profile are distributed onto curve elements and then mapped to the corresponding PRBM rigid bodies, serving as the external hydrodynamic inputs to the dynamic model of the robotic fish.

Regardless of the method used, an additional treatment is required to “softe” the rigid-body segments representing the flexible caudal peduncle, so as to better capture the continuous curvature characteristics of the actual flexible body. Specifically, the aforementioned “soften” treatment is implemented by taking the articulated joints as control points and employing a uniformly parameterized four-point Catmull-Rom algorithm to construct a cubic spline curve:

(20) $$P\left(t\right)=\frac{1}{2}\left[\begin{array}{cccc} 1 & t & t^{2} & t^{3} \end{array}\right]\left[\begin{array}{cccc} 0 & 2 & 0 & 0\\ -1 & 0 & 1 & 0\\ 2 & -5 & 4 & -1\\ -1 & 3 & -3 & 1 \end{array}\right]\left[\begin{array}{c} C_{0}\\ C_{1}\\ C_{2}\\ C_{3} \end{array}\right]$$

where $C_{i}$ is the control point, *t* is the interpolation parameter, and P(t) is the point on the spline curve which is also set as the ridge control line.

## 4. Performance Analysis

### 4.1. Vortex Analysis

**Figure 3:**
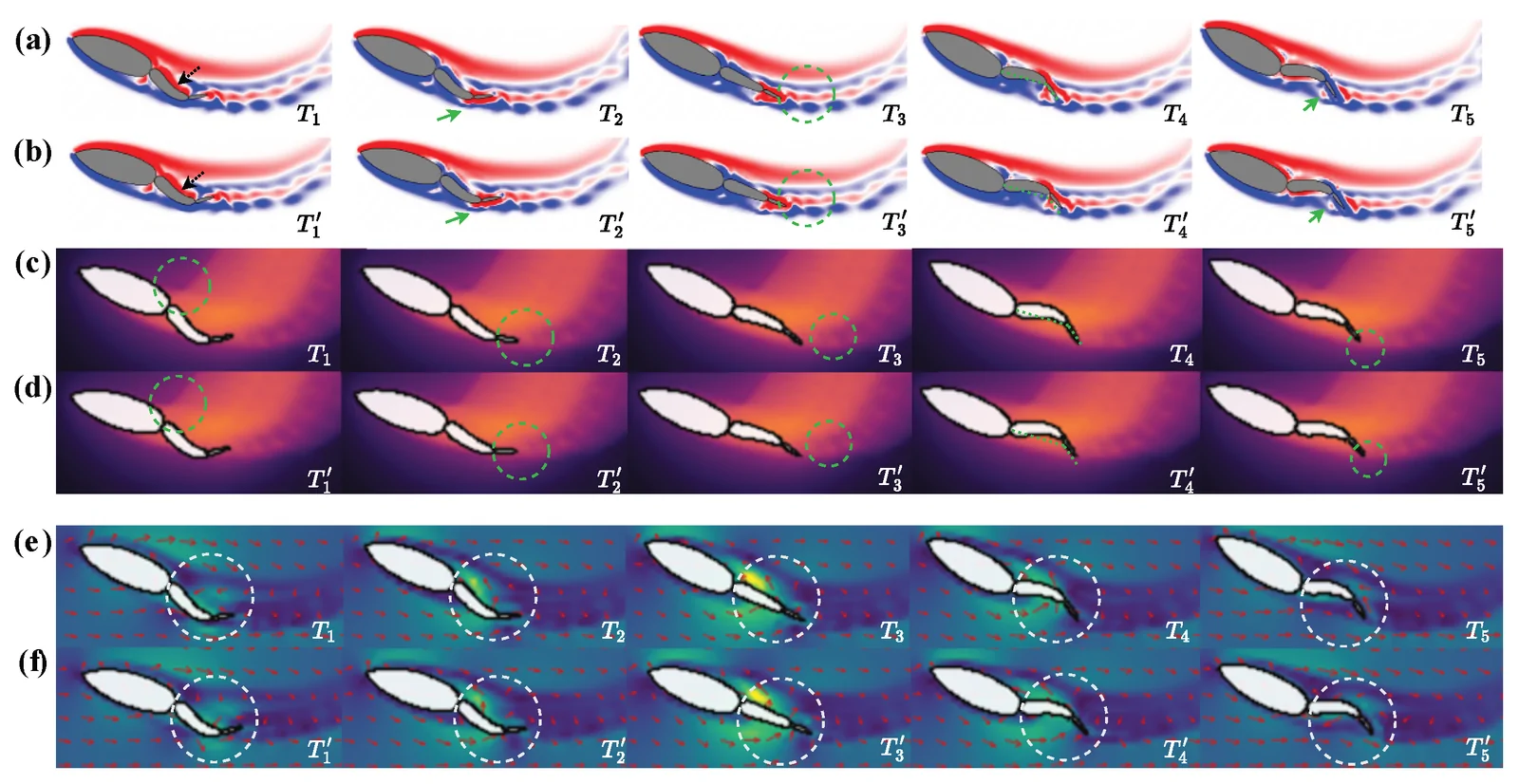
Vortex field distributions generated by the swimming robotic fish under different rotational inertia conditions. (**a**) Low rotational inertia condition (condition 1); (**b**) under high rotational inertia condition (condition 2); Turbulent kinetic energy distribution: (**c**) under condition 1, (**d**) under conditon 2; Velocity field: (**e**) under condition 1, (**f**) under condition 2.

Figure 3 presents the simulated fluid-structure interaction results of the PRBM-based robotic fish swimming under two different inertia configurations at a flapping frequency of 3 Hz. It can be observed that the cubic spline interpolation discretization adopted in the PRBM framework adequately captures the flexible characteristics of the caudal peduncle and produces satisfactory fluid-structure coupling interactions with the surrounding fluid. By comparison, clear discrepancies are observed in the bending profile of the caudal peduncle, the phase difference of the caudal peduncle, and the induced vortex structures as indicated by the circles and arrows in the figure. For instance, in Figure 3a,b, comparing $T_{1}/T_{1}^{\prime }$ reveals differences in the deformation profiles of the caudal peduncle segment, where the flexible caudal peduncle under high inertia exhibits a larger local deformation curvature. This discrepancy further leads to differences in the caudal peduncle phase, as indicated in the $T4/T^{\prime }4$ states. Meanwhile, we also observe that distinct inertia modes present certain differences in aspects such as fishtail-induced vortices and the reverse Kármán vortex street morphology of the tail jet, as illustrated in $T2/T^{\prime }2$, $T3/T^{\prime }3$, and $T5/T^{\prime }5$. In addition to the vorticity field, we also present the turbulent kinetic energy (TKE) distribution (Figure 3c,d) and the instantaneous velocity field contours (Figure 3e,f) during the fluid-structure interaction (FSI) process. It is evident that the coupling between the flexible caudal peduncle and the surrounding fluid differs substantially across inertia configurations. These differences are not only reflected in the deformation profile of the tail; they already manifest in the formation of leading-edge vortices near the anterior portion of the peduncle. As the flow evolves along the posterior region, the velocity fields diverge more markedly between the two inertia cases, ultimately leading to distinct wake structures in the rearward jet flow.

These findings collectively indicate that by adjusting the moment of inertia of the flexible fishtail, we can influence the bending profile of the flexible caudal peduncle and the vortex states in the flow field, thereby altering the interaction forces during the fluid-structure coupling process and subsequently affecting the swimming performance of the robotic fish.

### 4.2. Quantitative Simulation Analysis

**Figure 4:**
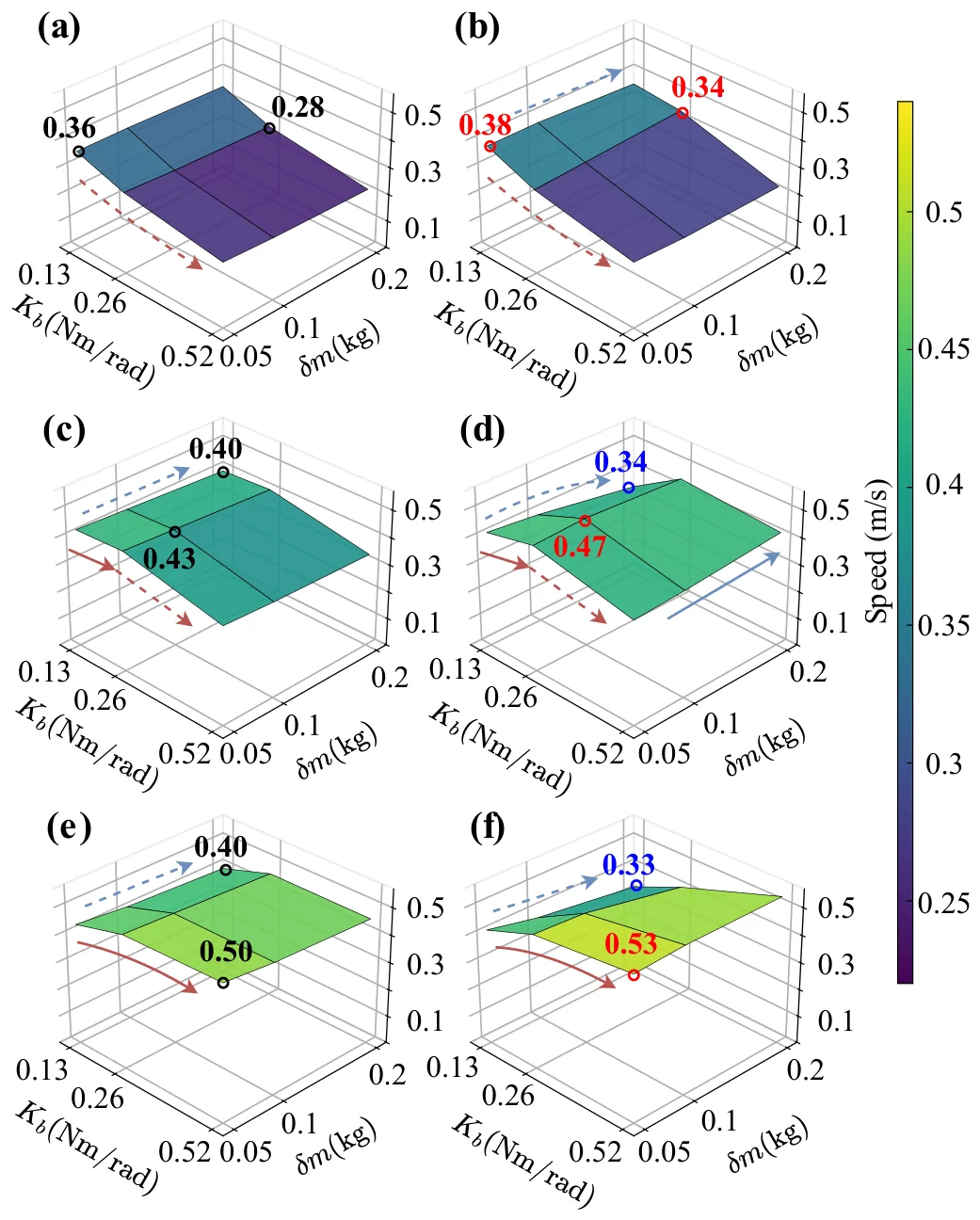
Effects of the two inertial tuning mechanisms on the average swimming speed of the robotic fish under varying tail-beat frequencies and flexible caudal peduncle stiffnesses. (**a**) 3 Hz with forward inertial mass configuration; (**b**) 3 Hz with rearward inertial mass configuration; (**c**) 4 Hz with forward inertial mass configuration; (**d**) 4 Hz with rearward inertial mass configuration; (**e**) 5 Hz with forward inertial mass configuration; (**f**) 5 Hz with rearward inertial mass configuration.

**Figure 5:**
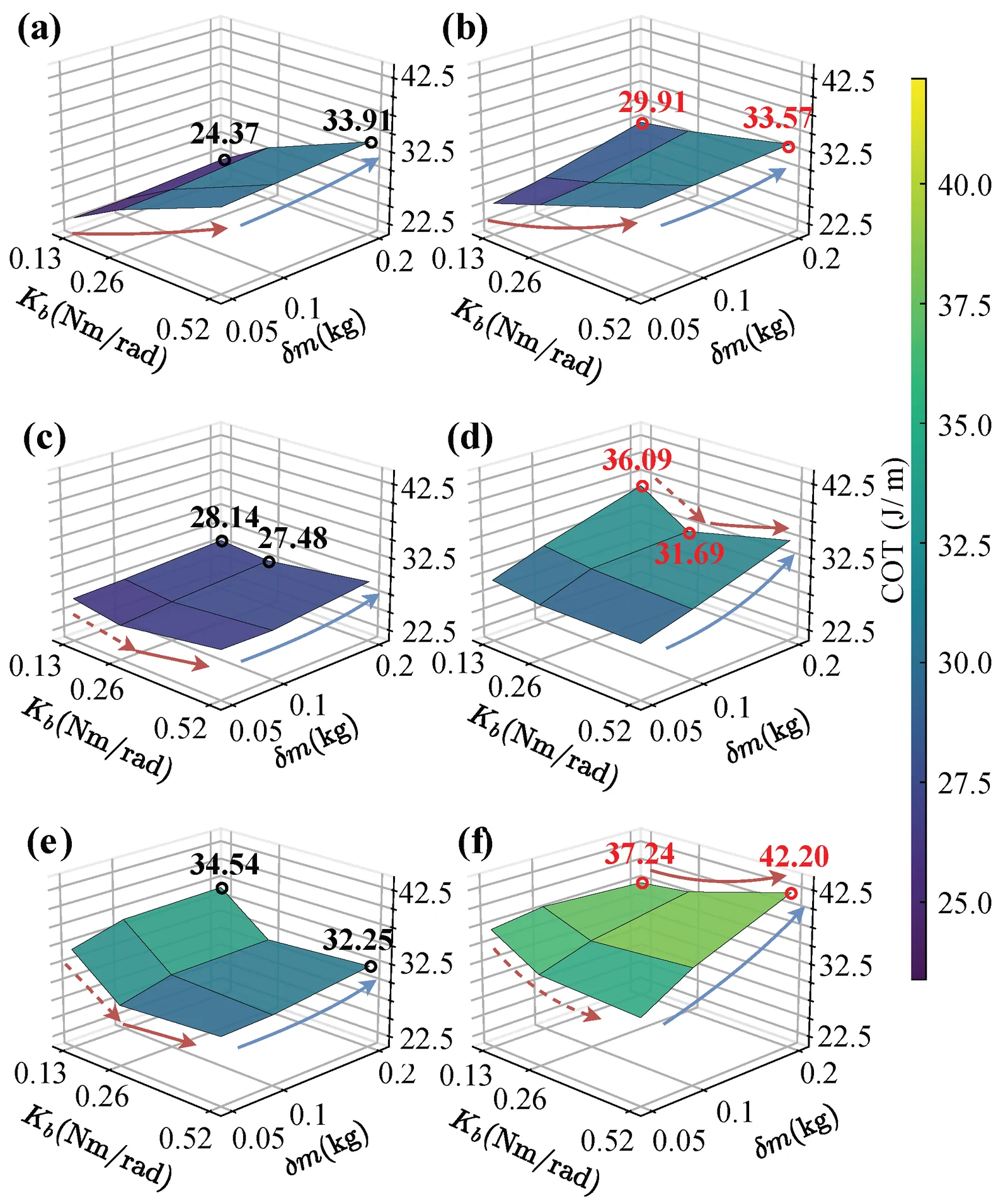
Effects of two inertia adjustment mechanisms on the COT of the robotic fish under varying tail-beat frequencies and flexible caudal peduncle stiffnesses. (**a**) 3 Hz with forward inertial mass configuration; (**b**) 3 Hz with rearward inertial mass configuration; (**c**) 4 Hz with forward inertial mass configuration; (**d**) 4 Hz with rearward inertial mass configuration; (**e**) 5 Hz with forward inertial mass configuration; (**f**) 5 Hz with rearward inertial mass configuration.

To more systematically investigate the effects of inertia adjustment on swimming performance, simulations over a wider parameter range were conducted, as shown in Figure 4 and Figure 5. The left column of each figure corresponds to the configuration with the inertia ball positioned at the front end of the caudal peduncle, while the right column corresponds to the rear-end configuration. From each subplot in Figure 4, it is evident that within the investigated parameter range, both the stiffness of the flexible caudal peduncle and the mass of the inertia ball affect the swimming speed. Since we focus on the effect of inertia, the stiffness is treated merely as a background variable. We found that increasing the rotational inertia by adding mass generally has a negative effect on swimming speed. A vertical comparison of Figure 5a,c,e (as well as b,d,f) reveals that as frequency increases, the effect of increasing the inertia ball mass becomes progressively more pronounced. This is especially true when the tail stiffness is low, in which case the mass increase leads to a notable decline in swimming speed.

An alternative approach to inertia adjustment is to keep the inertia ball mass unchanged while increasing the rotational inertia by repositioning the ball rearward toward the tip of the flexible caudal peduncle. A horizontal comparison between Figure 4a–c and Figure 4d–f reveals that increasing the rotational inertia can, in some cases, produce a distinctly positive effect on swimming speed. This effect is relatively consistent at low frequencies; for example, in many cases, increasing inertia from the configuration in Figure 4a to that in Figure 4b leads to an improvement in swimming speed. At higher frequencies, however, such as 4 Hz and 5 Hz, this beneficial effect becomes more complex, as seen by comparing Figure 4c–f. Furthermore, it is observed that when the caudal peduncle is relatively stiff, an increase in inertia tends to improve swimming speed, whereas when it is relatively soft, the same increase tends to reduce swimming speed.

### 4.3. Thrust Test and Comparison

**Figure 6:**
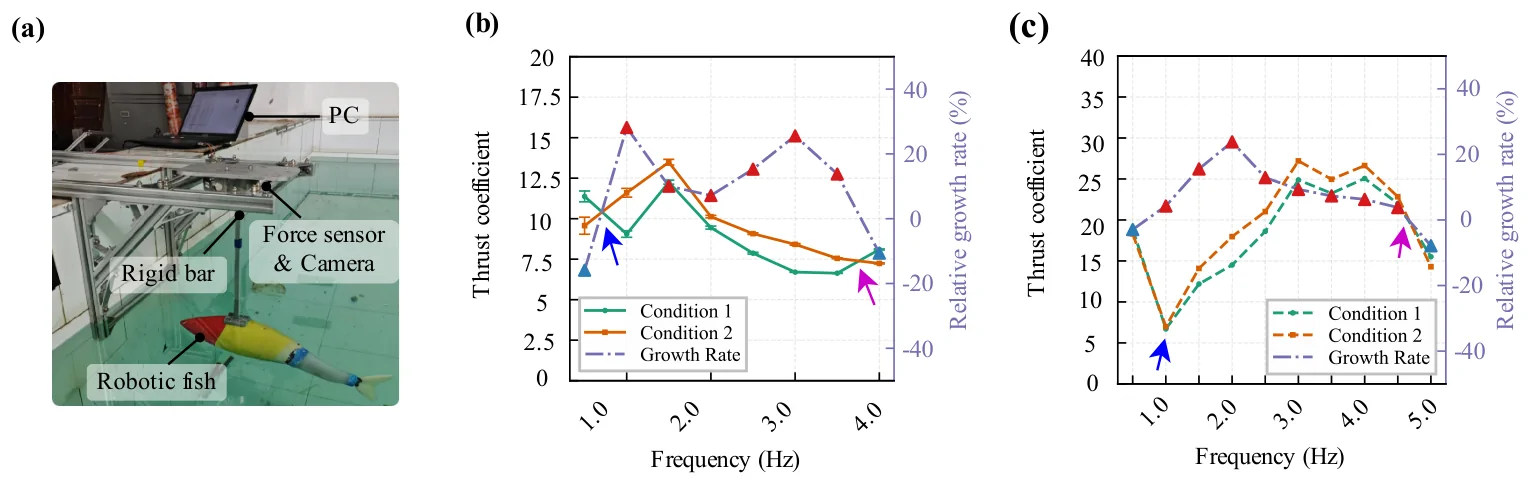
Comparison of robotic fish thrust performance under different inertia conditions. Condition 1: low inertia (inertia ball forward, $\delta I=20\;\mathrm{kg}\cdot {\mathrm{mm}}^{2}$); Condition 2: high inertia (inertia ball rearward, $\delta I=940\;\mathrm{kg}\cdot {\mathrm{mm}}^{2}$). (**a**) Experimental setup. (**b**) Test results. (**c**) Simulation results.

To verify the feasibility of the mechatronic design and the accuracy of the proposed dynamic models, a series of swimming experiments was conducted. However, because the online inertia adjustment system requires the inertia ball to be spherical and capable of moving freely within the caudal peduncle cavity, the experiments were restricted to a limited range of operating conditions. Figure 6 presents the thrust test results under different rotational inertia configurations. As illustrated in Figure 6a, the head of the robotic fish was rigidly linked to an ATI Delta IP6 NET six-axis force/torque sensor mounted on a test bench. The streamwise thrust signals captured by the force sensor were filtered and averaged to obtain mean thrust values and converted into the thrust coefficient $C_{T}^{*}$ across varying tail flapping frequencies. The definition of $C_{T}^{*}$ is as follows:

(21) $$C_{T}^{*}=\frac{T}{0.5\rho {\left(fA\right)}^{2}S}$$

where *T* denotes the thrust force, ρ denotes the fluid density, *f* denotes the flapping frequency, *A* denotes the peak-to-peak tail-tip amplitude and *S* represents the wetted surface area of the robotic fish. Figure 6b reveals that, at low flapping frequencies, the difference in mean thrust coefficient between Condition 1 (low inertia) and Condition 2 (high inertia) is marginal. As frequency increases (as shown by the blue arrow in the figure), Condition 2 produces higher average thrust coefficient. However, this performance advantage does not persist across all frequencies: at about 4 Hz (as shown by the pink arrow in the figure), the low-inertia condition surpasses the high-inertia condition in thrust output. Figure 6c illustrates the corresponding numerical simulation results for the two test cases, which also reveal evident thrust discrepancies induced by different inertia magnitudes. Condition 1 yields greater thrust coefficient at both low and high frequency ranges, while the high-inertia configuration achieves superior thrust performance within the intermediate frequency range. Note that the simulation conditions at 4.5 Hz and 5 Hz lack corresponding experimental data, as we did not run the robotic fish under these conditions for long periods to protect the mechanical components.

### 4.4. Free Swim Test and Comparison

**Figure 7:**
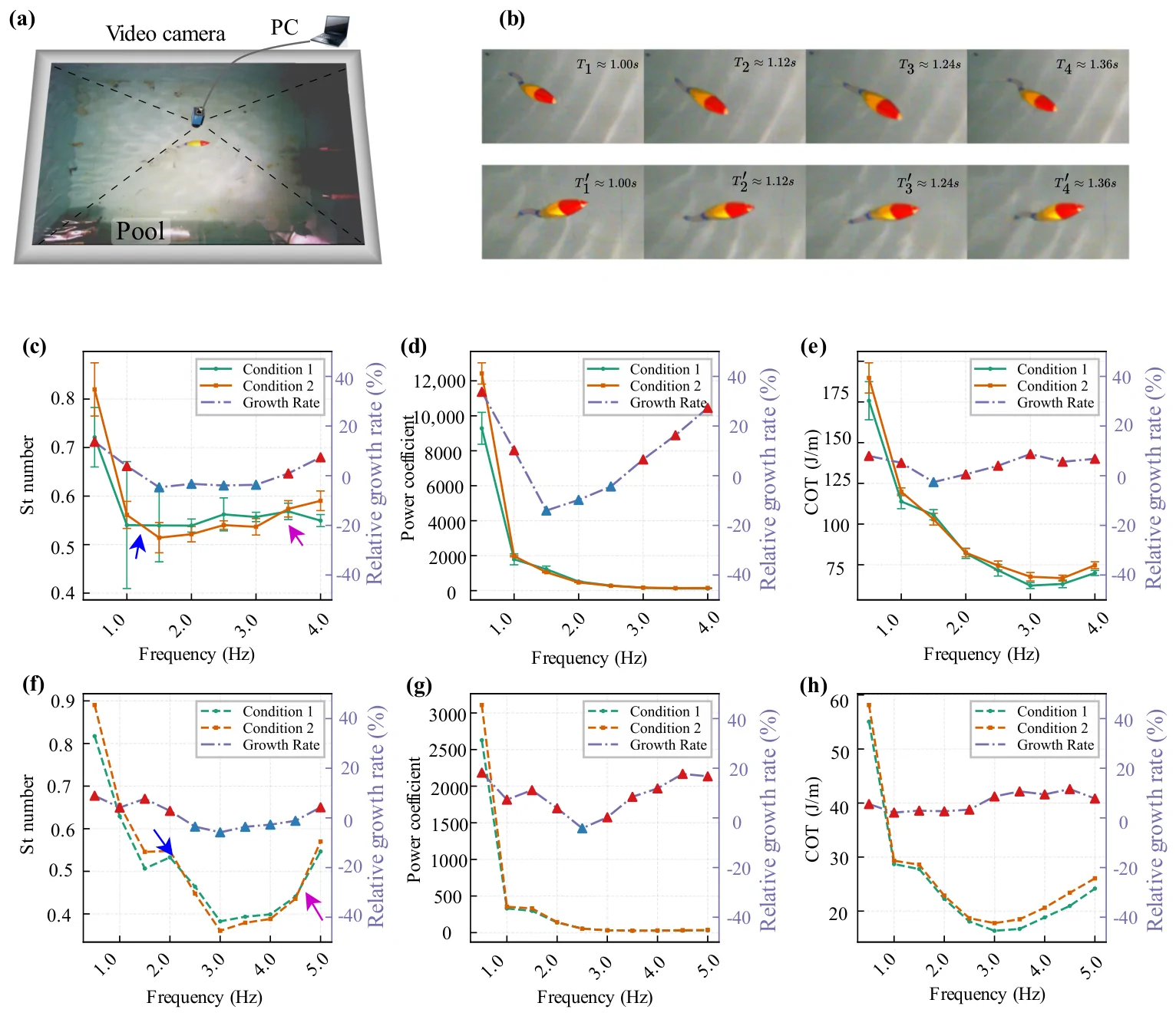
Comparison of robotic fish swimming performance under different inertia conditions. Condition 1: low inertia (inertia ball forward, $\delta I=20\;\mathrm{kg}\cdot {\mathrm{mm}}^{2}$); Condition 2: high inertia (inertia ball rearward, $\delta I=940\;\mathrm{kg}\cdot {\mathrm{mm}}^{2}$). (**a**) Experimental setup. (**b**) Swimming snapshots of the robotic fish under small/large moment of inertia at a flapping frequency of 3 Hz. Experimental results: (**c**) St number, (**d**) Average power consumption, (**e**) COT. Simulation results: (**f**) St number, (**g**) Average power consumption, (**h**) COT.

Comparative experiments were conducted to investigate the free-swimming performance of the robotic fish under different inertia configurations. To characterize the unsteady hydrodynamic status, we first calculate the Reynolds number for our system

(22) $$Re=\frac{VL}{\nu }$$

where *V* is the swimming velocity, *L* is the body length of robotic fish and ν is the kinematic viscosity of water at 20 °C. For free-swimming tests, these values ($0.68∼1.9\times 10^{5}$ within swimming frequency 1∼5 Hz) are consistent with the transitional/turbulent flow regime typically observed in BCF fish swimming. Figure 7a shows the experimental setup. An overhead camera was mounted above a water tank with dimensions of $5\times 4\times 1.2\;m^{3}$. Real-time target recognition was performed on the robotic fish, which was marked with red and yellow stickers, from which swimming velocity and other performance metrics were derived. Figure 7b presents sequential snapshots of the robotic fish over one undulation cycle. States $T_{1–4}$ correspond to the caudal peduncle with low rotational inertia, while states $T_{1–4}^{\prime }$ correspond to the high-inertia configuration. Figure 7c–e present the various swimming performance metrics of the robotic fish measured experimentally under different inertia conditions. The swimming velocity is normalized using the characteristic velocity associated with the tail-beat motion, namely Strouhal number:

(23) $$St=\frac{fA}{V}$$

where *V* is the forward swimming speed. It can be observed that in the low-frequency range, the flexible caudal peduncle with greater rotational inertia exhibits a higher St number. However, as the frequency increases, the St number under the low-inertia condition overtakes that under the high-inertia configuration, though the trend reverses again at 4 Hz. A similar pattern is observed for the power coefficient ($C_{p}$), which is defined as:

(24) $$C_{p}=\frac{P_{w}}{0.5\rho V^{3}S}$$

where $P_{w}$ is the power consumption. That means the high-inertia configuration exhibits a local advantage over the low-inertia case within the intermediate frequency range, while outside this range, however, the low-inertia condition yields a lower power coefficient. Notably, at low frequencies, both power consumption and COT exhibit anomalously higher values, which is attributed to mechanical friction within the system and baseline electrical losses. We further evaluate the energy efficiency using the cost of transport (COT), defined as:

(25) $$COT=\frac{P_{w}}{V}$$

The results show that increasing inertia consistently leads to higher energy expenditure. This may be one of the reasons why most biological fish possess a gradually tapered caudal peduncle. Figure 7f–h present the simulation results under the same operating conditions. All metrics exhibit nearly the same trends as the experimental values. In summary, for both thrust and swimming speed, increasing inertia enhances performance within a certain range of tail-beat frequencies. However, beyond this range, the effect of high inertia on the swimming performance of the flexible caudal peduncle shifts from beneficial to detrimental.

### 4.5. Discussion

In this work, a novel decoupled online-inertia-tunable robotic fish and a corresponding dynamic model integrating the PRBM and BDIM methods are developed. The swimming performance under inertia modulation is systematically evaluated through both numerical simulations and experimental tests. The results demonstrate that increased rotational inertia consistently increases energy consumption. This observation is qualitatively consistent with the tapered morphology of the caudal peduncle observed in many fish species, although the underlying biological mechanisms require further investigation. Nevertheless, increased inertia also presents distinct advantages. Within a specific tail-beat frequency range, higher inertia effectively improves thrust coefficient and swimming performance, with a maximum thrust coefficient enhancement of 26% (from 6.70 to 8.42 at 3 Hz) in experiments and 24% (from 14.51 to 17.97 at 2 Hz) in simulations, and a swimming performance improvement of 5% reduction of St number(from 0.54 to 0.51 at 2 Hz) in experiments and 6% in simulations (from 0.38 to 0.36 at 3 Hz). Comparative analyses with stiffness further verify that such performance benefits persist and is correlated with stiffness variations. Notably, at high tail-beat frequencies (4.5 Hz and 5 Hz), where increased inertia would otherwise exert adverse effects, increasing the tail stiffness can reverse this negative influence into a positive one. Specifically, the effect of inertia on swimming speed improves from −15% to 9.3% at 4.5 Hz and from −17.5% to 6% at 5 Hz. In addition, a comparative study of inertia tuning strategies reveals that, unlike the proposed mass-redistribution-based method, the direct strategy of directly increasing total mass at fixed positions almost always deteriorates both maneuverability and energy efficiency. To contextualize these findings within the broader state-of-the-art, Table 2 systematically compares our decoupled inertia modulation framework with existing variable-stiffness and variable-inertia mechanisms across various robotic disciplines. From a practical engineering perspective, the proposed decoupled inertia tuning mechanism provides a complementary actuation modality for robotic fish, distinct from traditional variable-stiffness tails. The proposed purely mechanical symmetric mass-redistribution mechanism provides a simple and effective approach to online inertia tuning. It enables the robotic fish to adjust its rotational inertia according to different swimming frequencies and amplitudes, thereby providing greater flexibility in adapting to different operating conditions and tasks. Meanwhile, it can complement traditional stiffness-based approaches by introducing an additional degree of freedom for regulating swimming performance.

Although the present study has clarified the phenomenological influences of inertia modulation on the thrust output, swimming speed, and energy consumption of robotic fish, the underlying hydrodynamic mechanisms require further in-depth exploration. Notably, rotational inertia increment can either improve or deteriorate locomotion performance in a frequency-dependent manner, yet the detailed vortex dynamics dominating such inertia-induced performance variations remain poorly understood. These unclarified key factors include the evolution of wake topology, spatial distribution of vorticity, and jet flow characteristics generated by tail flapping. Accordingly, further vortex-level fluid–structure interaction investigations are essential to comprehensively reveal how inertial tuning mediates momentum transfer between the flexible caudal peduncle and ambient fluid domains.

## 5. Conclusions and Future Work

In this study, a novel decoupled, online-inertia-tunable bionic robotic fish was designed, prototyped, and evaluated. Benefiting from a mirror-symmetric, cable-driven timing belt mechanism with synchronized mass spheres, the platform successfully achieves real-time, independent modulation of tail rotational inertia while keeping the global center of mass completely fixed. To evaluate this system, a high-fidelity fluid-structure interaction (FSI) dynamic model was established by integrating the pseudo-rigid-body method (PRBM) with the boundary data immersion method (BDIM). Both numerical simulations and physical experiments systematically demonstrated that rotational inertia acts as a effective independent control input that significantly governs the swimming kinematics, wake topology, and swimming performance of fish-inspired underwater robots. Moving beyond empirical data observations, several practical conclusions can be derived from this study. First, rotational inertia exerts a distinct frequency-dependent effect on robotic fish locomotion. Specifically, moderate inertial augmentation enhances thrust and swimming speed within a certain range of tail-beat frequencies, whereas the same augmentation impairs performance in other frequency regimes. Second, the energy consumption efficiency exhibits a monotonic decline with increasing rotational inertia across all tested working conditions. Furthermore, the functional outcomes of inertial tuning are strongly dependent on the modulation strategy. Simple direct mass addition consistently impairs overall swimming performance, while symmetric mass redistribution can effectively yield favorable locomotion improvements for bionic robotic fish.

In the future, we will focus on investigating the detailed vortex-level flow physics—such as spatial vorticity distribution, jet characteristics and variation of force component coefficients (drag, lift, moment etc) depending on angle of attack. Additionally, we aim to develop a fully autonomous, closed -loop adaptive inertial control system that dynamically optimizes internal mass positions based on real-time sensory feedback in changing aquatic environments. A robotic fish capable of simultaneous stiffness and inertia tuning will also be developed. This integrated approach is expected to provide greater flexibility in regulating swimming performance, and its applicability will be further validated in more complex underwater environments and task scenarios.

## Figures and Tables

**Table 1:** Main Parameters of the Developed Robotic Fish.

Attribute	Parameter
Dimension (L×W×H)	∼0.62×0.08×0.135 (m^3^)
Total mass	∼2.8 kg
Movable mass	∼0.045 kg
Power system	Li-ion battery (DC 32 V, 26 Ah)
Control system	STM32F407ZGT6 MCU (STMicroelectronics, Plan-les-Ouates, Switzerland)
Drive system	Tail flapping motor (Maxon EC-4 Pole 200 W) (Maxon Motor AG, Sachseln, Switzerland),
	Inertia regulation motor (DC-12T-N20) (Shenzhen JFT Technology Co., Ltd., Shenzhen, China)
Communication system	LoRa module HC-14 (Make Electronics(HC Tech), Guangzhou, China)

**Table 2:** Comparison of regulation mechanisms and key performance across robotic fields.

Mechanism	Key Performance Results	Tune Type	Research Field
Internal eccentric rolling mass [22]	· Suppresses low-speed roll · Stabilizes sonar attitude	Inertia	AUV
Retractable 4-DoF prismatic tail [23]	· Optimizes jump/landing · Prevents pitch tumbling via inertial torque	Inertia	Legged robots
Joint-coordinated inertia axes rearrangement [24]	· Propellant-free detumbling · Reduces post-capture coupling	Inertia	Space robotics
Vacuum-driven layer jamming muscles [18]	· thrust + 142% at 5 Hz	Stiffness	Robotic fish
Silicone-embedded SMA wire [28]	· Speed + 35.5%	Stiffness	Robotic fish
ERF with PCB-microfiber [15]	· Active tuning enables fast convergence to optimal St regime	Stiffness	Robotic fish
Hydraulic Variable Stiffness (HVS) [29]	· Turning radius ∼0.25 · Turning rate ∼3×	Stiffness	Robotic Fish
Ours	· $C_{T}^{*}$ + 26% · Approaching the St number of real fish	Inertia	Robotic fish

## Data Availability

The data generated during the current study are available from the corresponding author on reasonable request.

## Abbreviations and Symbols

**Abbreviations**	
AUV	Autonomous Underwater Vehicle
BCF	Body and/or Caudal Fin
BDIM	Boundary Data Immersion Method
COM	Center of Mass
COT	Cost of Transport
DoF	Degree of Freedom
ERF	Electrorheological Fluid
FSI	Fluid-Structure Interaction
MCU	Microcontroller Unit
PRBM	Pseudo-Rigid Body Method
SMA	Shape Memory Alloy
TKE	Turbulent Kinetic Energy
UAV	Unmanned Aerial Vehicle
**Symbols**	**Definition**
*A*	Peak-to-peak tail-tip amplitude
a(z)	Major-axis length at *z*
*B*	Bending stiffness
b(z)	Minor-axis length at *z*
C	Coriolis-centrifugal matrix
$C_{a}$	Added-mass coefficient
$C_{d}$	Pressure drag coefficient
$C_{f}$	Viscous resistance coefficient
$C_{P}$	Power coefficient
$C_{T}^{*}$	Thrust coefficient
$D_{i}$	Wetted height of *i*-th link
*E*	Young’s modulus
*f*	Tail-beat frequency
G	Conservative forces
$I_{i}$	Inertia of *i*-th link
$J_{v,i}$	Linear velocity Jacobian
$J_{\omega ,i}$	Angular velocity Jacobian
$k_{i}$	*i*-th joint stiffness
$K_{k},K_{b}$	Stiffness distribution coeffs
M	Inertia matrix
$m_{i}$	Mass of *i*-th link
$M_{fd}$	Coupling inertial coefficient
$P_{w}$	Average power consumption
q	Generalized coordinates
$r_{1},r_{2}$	Operational lengths op, $o^{\prime }p$
Re	Reynolds number
$r_{i}$	Position vector of *i*-th centroid
$r_{l,i}$	Vector: joint to *i*-th centroid
$r_{t,i}$	Vector: *i*-th centroid to distal joint
*S*	Wetted surface area
St	Strouhal number
*s*	Curvilinear coordinate
t(z)	Wall thickness at *z*
*T*	Total kinetic energy / Thrust force
*U*	Elastic potential energy
u,v	Planar coordinates of first link
${\hat{u}}_{i}$	Unit tangential vector
*V*	Forward swimming velocity
$v_{⊥,i},v_{\|,i}$	Normal/tangential velocities
${\hat{w}}_{i}$	Unit normal vector
$\delta I_{i}$	Added rotational inertia
$\delta m_{i}$	Added mass
θ	Motor rotation angle
$\theta_{i}$	Absolute deflection angle
ν	Kinematic viscosity
$\xi_{1},\xi_{2}$	Geometric constraint angles
ρ	Fluid density
$\phi_{k}$	Relative joint angle
$\phi_{2}$	Fishtail swing angle
